# Biocompatible Hybrid Surface Layers on Porous Magnesium Structures Fabricated by Spark Sintering

**DOI:** 10.3390/jfb16080269

**Published:** 2025-07-22

**Authors:** Konstantine V. Nadaraia, Anastasia A. Golysheva, Evgeniy A. Belov, Dmitry A. Lyapin, Mariia S. Gerasimenko, Maria A. Nadaraia, Arina I. Pleshkova, Igor M. Imshinetskiy, Oleg O. Shichalin, Anton A. Belov, Eugeniy K. Papynov, Sergey S. Atarshchikov, Dmitry V. Mashtalyar

**Affiliations:** 1 Institute of Chemistry FEB RAS, 159 Pr. 100-letiya Vladivostoka, Vladivostok 690022, Russia; belov_eal@mail.ru (E.A.B.); gerasimenko.ms00@mail.ru (M.S.G.); nadaraia.maria.al@gmail.com (M.A.N.); othariadna@gmail.com (A.I.P.); igorimshin@gmail.com (I.M.I.); madiva@inbox.ru (D.V.M.); 2 Far Eastern Federal University, 10 Ajax Bay, Russky Island, Vladivostok 690922, Russia; anastasiagolysheva2003@gmail.com (A.A.G.); lyapin.da@dvfu.ru (D.A.L.); shichalin_oo@dvfu.ru (O.O.S.); belov_aa@dvfu.ru (A.A.B.); papynov.ek@dvfu.ru (E.K.P.); 3 Sakhalin State University, Communist Ave., 33, Yuzhno-Sakhalinsk 693008, Russia; 4 Medical Center, Far Eastern Federal University, 10 Ajax Bay, Russky Island, Vladivostok 690922, Russia; atarshchikov.ss@dvfu.ru

**Keywords:** magnesium alloy, plasma electrolytic oxidation, spark plasma sintering, hydroxyapatite, vancomycin, menaquinone MK-7, zoledronate, polydopamine

## Abstract

In this study, 3D Mg scaffolds were obtained by the spark plasma sintering (SPS), and a calcium phosphate coating was then obtained on the samples by the plasma electrolytic oxidation. A hybrid coating with vancomycin, zoledronic acid, and menaquinone MK-7 was formed to improve biocompatibility. The mechanical properties of the formed specimens were studied. According to XRD, XRF, SEM, EDS, and OSP studies obtained scaffolds have developed morphology and contain hydroxyapatite as well as bioactive substances. Formation of coatings improves the wettability of samples (contact angle decreases from 123.8 ± 3.1° to 26.9 ± 4.1°) and increases the surface roughness by more than 3 times. This makes them promising for use as a new generation of implantation materials. The results are important for the development of personalized implants with improved functional characteristics.

## 1. Introduction

Currently, magnesium-based alloys are one of the most promising implantation materials in orthopedic applications due to their numerous advantages, including a low weight of the final product, a low cost of manufacturing, bioresorbability, and biocompatibility [1,2,3]. However, challenges still exist, such as inconsistencies between the implants and the anatomical features of patients, reduced cell adhesion to the implants, and implant-associated infections (IAIs).

The solution to this problem lies in the use of additive technologies. Their key strength is the ability to produce complex, specialized structures with high precision. The application of these methods ensures superior control over the material, enabling the production of components with enhanced mechanical properties [4,5].

The spark plasma sintering (SPS) can be utilized to create three-dimensional (3D) samples, allowing for personalized implants. This not only enhances the reliability of the products but also accelerates the recovery process [6]. Thus, previously, the spark plasma sintering method was successfully used for the synthesis of porous ceramic materials, including biocompatible frameworks [7] and corrosion-resistant implants [8], as well as for the novel synthesis of nano-Hap with controlled morphology [9]. This confirms the promise of using SPS for the development of porous ceramics with controlled degradation in vivo.

Additionally, plasma electrolytic oxidation (PEO), a coating formation method based on creating plasma microdischarges followed by rapid cooling of the affected area, can help solve the issue of cell adhesion. As a result of the PEO process, a porous structure is formed, which helps to increase the surface area of contact between the implant and surrounding tissues. This has a positive impact on cell adhesion to the implant’s surface [10].

Hydroxyapatite (HA), the main mineral component of bone tissue, contains calcium and phosphate ions, which accelerate the formation of callus. This makes HA an excellent synthetic material for replacing lost bone tissue [11]. One way to form HA on the PEO-coated surface is by adding specific substances to the electrolyte solution. According to the literature, various modifications of the electrolyte can be used for the synthesis of compounds during the PEO process [12,13]. Calcium glycerophosphate (CaPO_4_C_3_H_7_O_2_) is often used to create HA-based coatings [14,15,16,17]. During oxidation, calcium glycerophosphate decomposes into calcium and phosphate ions, which then form calcium orthophosphate, the crystallization precursor of HA. The concentration of calcium glycerophosphate in the electrolyte significantly influences the HA content in the resulting coating layers.

To address the issue of IAIs, studies are being conducted to incorporate antibiotics into the porous structure of the coating [18]. Additionally, it is possible to create heterolayered structures that facilitate the gradual release of drugs with osteogenic (menaquinone-7, MK-7) [19,20], antibacterial (vancomycin) [21], and antitumor (zoledronate) properties [22,23]. To ensure a controlled, layered release of these substances, it is proposed to develop hybrid polymer-containing layers using polydopamine (PDA). Due to its excellent biocompatibility, ease of polymerization, anti-inflammatory properties, and ability to promote HA deposition, PDA is widely used for forming implant surface coatings and creating nanomaterials [24,25,26].

In summary, the presented study aims to develop a new generation of implants that combine a wide range of critical properties and enable targeted drug delivery.

## 2. Materials and Methods

The synthesis of the matrices was performed using SPS by consolidating the powders on an SPS-515S unit (Dr. Sinter·LAB™, SPS Syntex Inc., Kyoto, Japan). To improve the biocompatibility of the resulting samples, a powder mixture of magnesium and calcium carbonate with a total mass of 2 g was used. The mass concentration of CaCO_3_ was 0, 1, 2, 3, 5, 7, 10 wt.%, respectively. The powder consolidation was carried out using SPS at a temperature of 550°C, under a pressure of 30 MPa. The sample was kept at the maximum temperature for 5 min, then cooled to room temperature over a period of 30 min. The resulting cylindrical matrix samples had a diameter of 20 mm and a height ranging from 3 to 4 mm.

In this study, the mode of PEO was selected based on previous research into the creation of bioactive coatings on magnesium alloys [24]: a bipolar mode ensured the formation of not only α-Ca_3_(PO_4_)_2_ on the coating surface, which acts as a center for the crystallization of HA, but also directly HA. PEO was carried out in an electrolyte solution consisting of CaPO_4_C_3_H_7_O_2_ (25 g/L), NaF (5 g/L), and Na_2_SiO_3_ (7 g/L). During the PEO process, the voltage was increased from 50 V to 400 V for 100 s to form plasma microdischarges at the interface between the electrolyte/natural oxide film, which discharges are necessary for the formation of the PEO layer. The duration of the PEO process was 100 s. The thickness of the formed PEO coatings was 70–90 µm [27]. All samples were washed with distilled water, degreased with alcohol, and dried with warm air.

**Figure 1:**
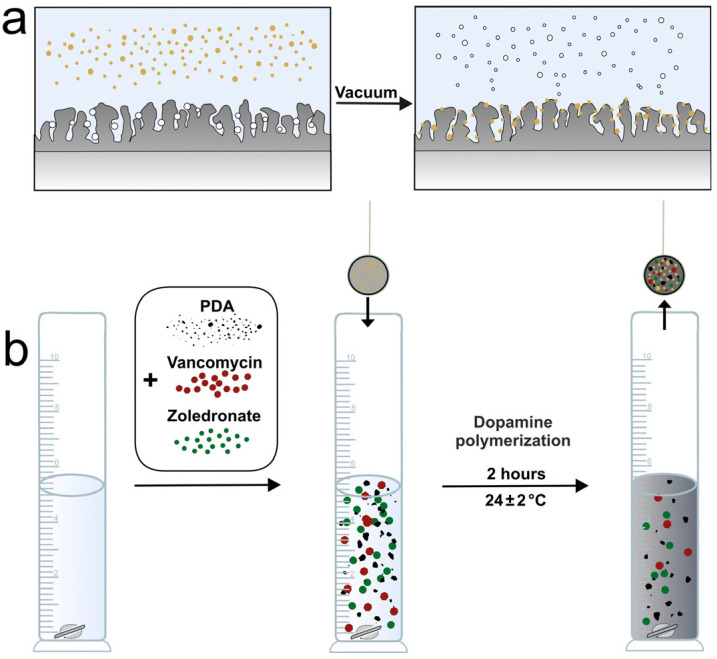
Illustration of hybrid coating preparation process, (**a**)—impregnation of menaquinone-7, (**b**)—polymerization of dopamine (formation of hybrid layer).

The formation of the hybrid layer occurred in 2 stages. In the first stage, menaquinone-7 was dissolved in 95% alcohol at a concentration of 0.15 mg/mL and then impregnated into the PEO layer under low-pressure conditions (0.95 mbar) (Figure 1a).

Next, a polymerization solution was prepared according to the protocol presented in our previous paper [27]. Dopamine (3 mg/mL), vancomycin (5 mg/mL), and zoledronate (0.03 mg/mL) were dissolved in 10 mM TRIS-HCl buffer (pH 8.5, Sigma-Aldrich, St. Louis, MO, USA) (Figure 1b). Samples with a pre-applied layer of menaquinone-7 were kept in separate test tubes with 5 mL of solution for 2 h at room temperature. The treated samples were removed from the test tubes, washed with deionized water, and air dried. The designation of prepared samples is presented in Table 1.

X-ray diffraction (XRD) analysis was performed using a D8 Advance X-ray diffractometer (Bruker, Karlsruhe, Germany), covering a 2θ angular range from 5° to 80° with a step size of 0.02° and an exposure time of 1 s/point. Phase identification was carried out using the “EVA” search-match program.

The elemental composition of samples was determined using X-ray fluorescence analysis on an EDX-800HS energy dispersive spectrometer (Shimadzu, Kyoto, Japan).

The surface morphology and elemental composition of the samples were studied using an EVO 40 scanning electron microscope (SEM) (Carl Zeiss, Oberkochen, Germany) equipped with an INCA X-act energy dispersive spectroscopy (EDS) system (Oxford Instruments, Abingdon, UK). Before analysis, a thin layer (100 nm) of Cr was sprayed onto the samples.

The morphology and microrelief of the surface were analyzed using the OSP370 optical surface profiling technique on an M370 workstation (Princeton Applied Research, Oak Ridge, TN, USA). The scanning rate was 150 µm per second, with increments of 1 µm. Data analysis was conducted using Gwyddion 2.45 software (Department of Nanometrology, Czech Metrology Institute, Brno, Czech Republic). Surface roughness was quantitatively evaluated using three-dimensional topography parameters, specifically the arithmetic mean deviation (Sa) and root-mean-square deviation (Sq), where Sa indicates the average height variation across the surface and Sq represents the standard deviation of surface heights relative to a reference plane.

Surface wettability was determined using the sessile drop method with a DSA100 drop shape analyzer (Krüss, Hamburg, Germany). Simulated body fluid (SBF) was used as a test liquid in addition to the distilled water. Contact angle calculation was carried out using the Young–Laplace method [28]. The surface free energy (SFE) was calculated by the Owens-Wendt-Rabel-Kaelble (OWRK) method [29]. For the determination of contact angle (CA), deionized water and CH_2_I_2_ were used since the surface tension of these two liquids was established with high accuracy [30]. The volume of the test liquid was 5 µL during all measurements. The value of the CA was registered after the drop stabilized on the surface for 30 s. SFE was calculated according to Equation (1):

(1) $$\gamma_{s}=\gamma_{s}^{d}+\gamma_{s}^{p},$$

where *γ*ₛ is an SFE of solid phase, with *d* and *p* superscripts indicating the dispersive (van der Waals) and polar (dipole-dipole/hydrogen bonding) components

*γ_s_^d^* and *γ_s_^p^* were determined graphically from the linearized Owens equation [29], in which the intersection with the ordinate axis corresponds to the root of the dispersed components, and the slope of the straight line corresponds to the root of the polar components of the SFE of a solid phase (Equation (2)).

(2) $$\gamma_{l}\frac{1+\cos\left(\theta \right)}{2\sqrt{\gamma_{l}^{d}}}=\sqrt{\gamma_{s}^{d}}+\sqrt{\gamma_{s}^{p}}\sqrt{\frac{\gamma_{l}^{p}}{\gamma_{l}^{d}}},$$

where γl represents the surface tension of the liquid, with superscripts *d* and *p* denoting the dispersive (non-polar) and polar components of the liquid’s surface tension, respectively.

Tribological characteristics of the studied samples were measured at the TRB-S-DE unit (CSM Instruments, Peseux, Switzerland). Using this device, wear, friction coefficient, abrasion time, and number of revolutions are measured. The tests were carried out at a load of 5 N and a friction speed of 10 mm/s. The track diameter was 5 mm, and the number of cycles was 150. A corundum ball with a diameter of 5 mm was used as a counterbody.

Data are presented as mean ± SD (*n* ≥ 5). Errors in calculated parameters were determined through error propagation analysis of the measurement uncertainties.

## 3. Results and Discussion

### 3.1. SPS Process Characterization

**Figure 2:**
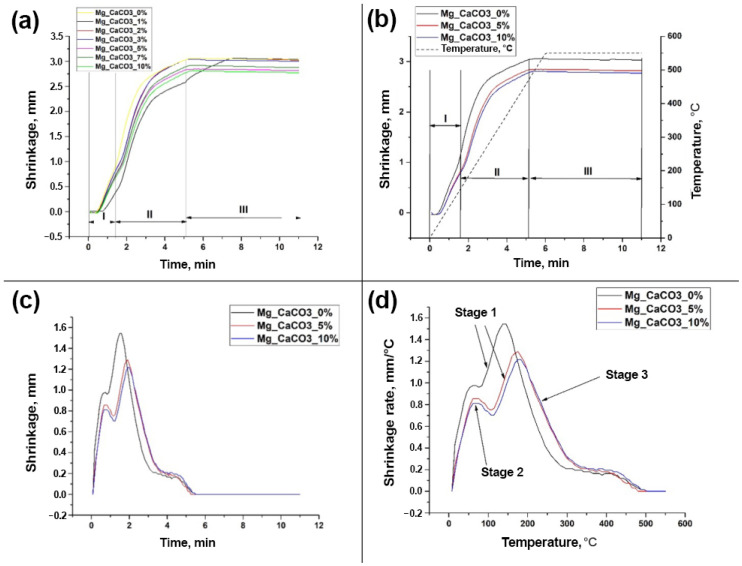
Dilatometric curves: overall shrinkage of the samples (**a**); shrinkage for samples containing different amounts of CaCO_3_ (**b**); time-dependent shrinkage rate for samples containing different amount of CaCO_3_ (**c**); temperature-dependent shrinkage rate for samples containing different amount of CaCO_3_ (**d**).

As a result of the SPS, the following dilatometric dependencies were obtained (Figure 2). All samples are characterized by a general compaction dynamic, which is determined by CaCO_3_, as shown in Figure 2. Powder compaction occurs in stages due to mechanical and thermal deformations.

Stage 1 is common to all samples and represents the mechanical compaction of the initial powder under pressing pressure (Figure 2a,b). Stage 2 is influenced by thermal heating and is accompanied by thermal compaction. According to the curves, the initial sintering stage (beginning of bending) occurs at a temperature of approximately 100 °C and reaches a plateau at 500 °C (Figure 2c,d). Stage 3 represents a plateau during which there are no significant changes in material compaction.

A similar trend was observed in the relationship between the shrinkage rate and temperature (Figure 2d). The temperature range between 100 and 200 °C corresponds to the main stage of material compaction, accompanied by a significant shrinkage rate. The range between 200 and 550 °C is characterized by a gradual decrease in the shrinkage rate and a plateau in shrinkage. Based on these findings, it can be concluded that an increase in the CaCO_3_/Mg ratio leads to a reduction in the absolute amount of shrinkage in the material and a decrease in its rate of change.

### 3.2. Coating Morphology

Next, the morphological characteristics of the resulting surface were investigated. This is an essential aspect in the development of bioactive coatings for medical implants. According to several studies [31,32,33], surfaces with a well-developed microstructure can improve cell adhesion. These investigations have identified the optimal surface roughness for implants, which falls within the *Ra* range of approximately 2.4–3.0 µm [34,35].

**Figure 3:**
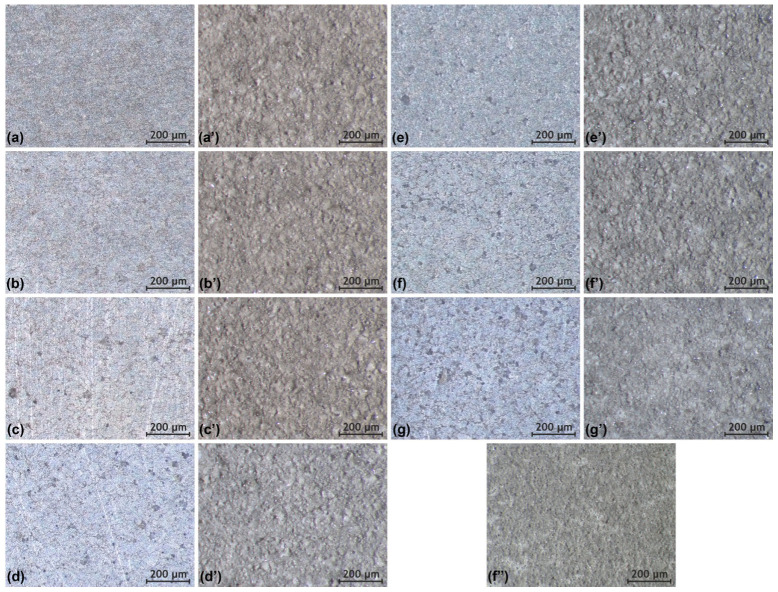
Optical images of SPS samples with different amounts of calcium carbonate in the coating after carbonic acid etching: (**a**)—0 wt.%, (**b**)—1 wt.%, (**c**)—2 wt.%, (**d**)—3 wt.%, (**e**)—5 wt.%, (**f**)—7 wt.%, (**g**)—10 wt.%; as well as PEO coatings obtained on these samples: (**a’**)—0 wt.%, (**b’**)—1 wt.%, (**c’**)—2 wt.%, (**d’**)—3 wt.%, (**e’**)—5 wt.%, (**f’**)—7 wt.%, (**g’**)—10 wt.%. Sample with hybrid layer: (**f”**)—7 wt.%.

Samples obtained by the SPS method were treated with a weak carbonic acid solution before applying the PEO coating to remove calcium carbonate particles and achieve a more developed surface morphology. Based on the analysis of the collected data, there is a trend of increased surface roughness in the coating as the proportion of calcium carbonate in the sample material composition increases. For instance, a sample containing 5 wt.% calcium carbonate, after treatment with the etching solution, exhibits a more developed and visually distinct morphology compared to samples with lower carbonate content, as observed in the microstructure of the PEO-coated surface (Figure 3a–e,a’–e’).

Samples containing 7 wt.% calcium carbonate exhibits a relatively more developed surface with a large number of pores and irregularities (Figure 3f). Based on the observed porosity, which was superior to other samples (Figure 3), it was decided to conduct further studies based on samples with 7 wt.% calcium carbonate proportion.

Samples containing 10 wt.% calcium carbonate have an even more developed surface due to the presence of pores and irregularities resulting from etching (Figure 3g). It was expected that the coating formed on these samples would exhibit a more complex morphology compared to samples with 5 wt.% and 7 wt.% calcium carbonate. However, the resulting PEO coating has a smoother surface texture than those on samples with lower calcium carbonate content (Figure 3d’,g’). This is likely due to the larger surface area of samples containing 10 wt.% calcium carbonate. Under the same coating formation conditions, a reduction in current density was observed, leading to fewer and less intense microdischarges, resulting in coatings with a less complex morphology.

The SPS-HYB sample does not visually differ from the SPS-PEO sample (Figure 3f’). The surface of the composite coating is not smooth and contains numerous irregularities; due to this, it has a positive effect on cell adhesion, as demonstrated in a previous study [31].

To confirm the conclusions drawn from our analysis of optical images, the morphology of SPS samples and samples with PEO coatings was examined using a laser profilometer (Table 2, Appendix A.1 and Appendix A.2). The increase in surface roughness of the samples after PEO coating formation is attributed to the high heterogeneity of the resulting layers. An increase in calcium carbonate concentration further leads to a significant rise in surface roughness. This behavior is explained by the presence of numerous non-conductive regions (CaCO_3_), which cause uneven distribution of plasma microdischarges across the sample surfaces.

According to the analyzed data, the 7% calcium carbonate samples exhibited maximal surface roughness. These samples developed a hybrid coating, which was selected for further investigation.

**Figure 4:**
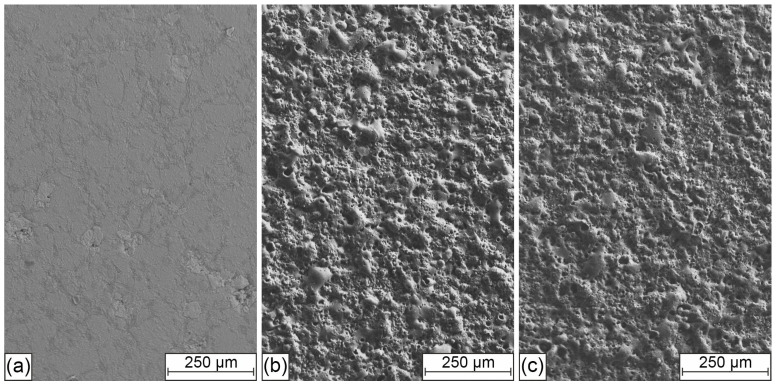
SEM images of the surface of bare SPS samples (**a**), with PEO coating (**b**), and with hybrid coating (**c**).

Evaluation of the SEM image of the uncoated sample indicates the presence of an uneven surface on which calcium inclusions are clearly visible (highlighted by arrows, Figure 4). The presence of calcium carbonate in the sample will additionally increase the rate of bone tissue regeneration during implant degradation.

Analysis of SEM images of the surface reveals that the base PEO coating on magnesium has a significant number of pores and other microdefects (Figure 4), caused by several factors. During the process, significant gas evolution occurs, and the breakdown zone rapidly cools to the electrolyte temperature as the plasma microdischarges weaken [36]. As a result, molten material solidifies at locations where powerful plasma discharges occur, forming pores. At first glance, the morphology of hybrid coatings appears similar to that of PEO coatings. However, a thin layer of polydopamine containing various bioactive components can be observed on the surface of hybrid-coated samples (Figure 4).

### 3.3. Coatings Composition

XRD data of SPS-PEO samples revealed that the presence of calcium carbonate in the magnesium substrate composition did not affect the phase composition of the coating (Appendix A.3) [31]. The main components of the PEO layer were magnesium oxide and magnesium orthosilicate, as well as hydroxyapatite. The presence of a small amount of amorphous phase (2θ = 5–15°) might be associated with the formation of numerous intermediate calcium phosphate compounds.

To check the composition and ratio of elements in quantity in the formed layers, X-ray fluorescence (XRF) was carried out. Due to this, it was possible to see the ratio of calcium and phosphorus in the composition of the coatings, which confirmed the formation of hydroxyapatite on the surface (Table 3).

Based on the results of XRF data, high levels of calcium were observed on the surface of the hybrid coating, accounting for more than a quarter of the total element composition. This calcium is predominantly present in the form of hydroxyapatite (Appendix A.3). However, an excess of phosphorus relative to calcium suggests the presence of other phosphate compounds such as magnesium-substituted hydroxyapatite and magnesium phosphate (Table 2). This, along with the presence of orthosilicate and magnesium oxide in the, explains the observed excess of magnesium [31]. The presence of orthosilicate is confirmed by the detection of silicon in the layer composition (Table 2). It should be noted that the analysis of hybrid coatings was challenging due to hardware limitations, which prevented the detection of elements with an atomic mass below 23. Carbon, nitrogen, oxygen, and chlorine, present in the organic components of the hybrid coatings, occur in such low concentrations that they are detected by the instrument with an error margin not exceeding 1%. However, in a previous study, samples with close surface composition were analyzed using Raman spectroscopy as well as X-ray photoelectron spectroscopy and IR spectroscopy [31].

**Figure 5:**
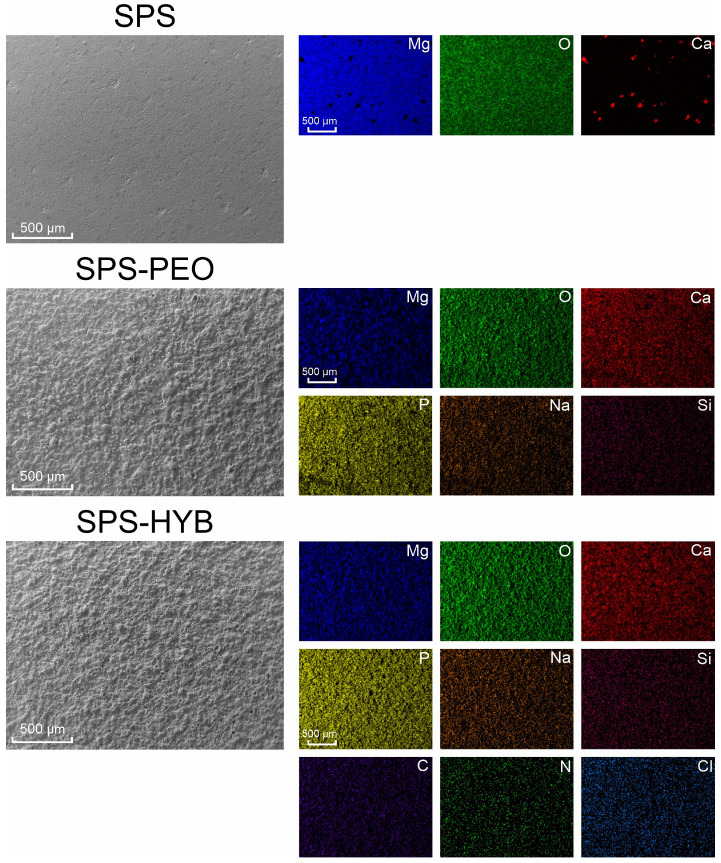
The results of EDS of bare SPS samples, with PEO, and with hybrid coating.

The EDS analysis is presented in Figure 5. For an uncoated sample, a high content of magnesium, oxygen, and calcium is observed, which are components of the powder material. On the surface of PEO-coated samples, elements present in both the sample and the electrolyte, such as magnesium, oxygen, sodium, calcium, and phosphorus, are evenly distributed. The hybrid coating SPS-HYB contains elements that are components of both the base PEO coating and the bioactive compounds (vancomycin, menaquinone-7, zoledronate, PDA). Taken together, this allows one to conclude that these compounds have been successfully incorporated into the surface layer.

### 3.4. Coatings Wettability and Surface Free Energy

**Figure 6:**
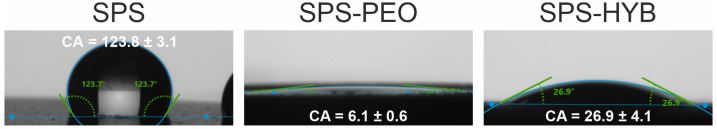
The shape of the water drops on different samples and CAs value.

**Figure 7:**
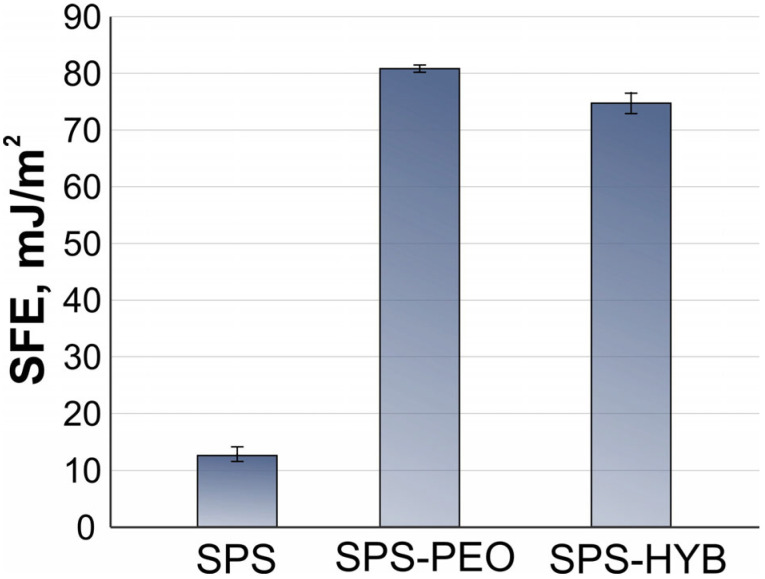
The samples’ surface free energy.

The obtained samples were also analyzed for surface wettability. The SPS sample exhibits hydrophobic properties, with a contact angle (CA) value of 123.8 ± 3.1° (Figure 6). However, it demonstrates a relatively low surface free energy (SFE) of 12.9 ± 1.7 mJ/m^2^ (Figure 6, Table 4). The formation of PEO coatings leads to a significant increase in wettability, attributed to two factors. First, water adsorption on oxide surfaces is significantly higher than on metal surfaces [37]. Second, PEO coatings have a highly developed surface structure, increasing the contact area between the liquid and solid phases (Figure 4) [38]. Additionally, the PEO layer has a well-developed porous structure, allowing droplets to penetrate deep into the coating [39]. As a result, the CA value on the SPS-PEO surface decreased by a factor of 20 (Figure 6), while the SFE value increased by a factor of 6 (Table 4, Figure 7).

The SPS-HYB sample showed values of CAs 26.9 ± 4.1°, indicating moderate hydrophilicity due to the presence of a PDA film and water-soluble components, such as vancomycin and zoledronate, in the hybrid coating. This led to a slight decrease in SFE value to 76.6 ± 3.2 mJ/m^2^ (Figure 7, Table 4). The results confirm that the use of PEO coatings and hybrid layers significantly improves the wettability of surfaces, which is crucial for improving biocompatibility and cell adhesion in biomedical implants.

There are studies that present the dependence of cell adhesion and proliferation on the polar component of the SFE of implants [40,41]. Thus, in study [42], implants with a high polar component of SFE on their surface demonstrated the highest density of adherent cells, while those with a low polar component showed higher expression of inflammatory mediators and lower cell proliferation. Based on this information, it can be inferred that received coatings on magnesium possess the highest values for the polar component of SFE, as shown in Table 4. Consequently, cell adhesion to such surfaces would be significantly higher than to uncoated samples.

The results obtained using deionized water are consistent with studies on the wettability of materials conducted using a simulated body fluid (SBF). SBF approximates human blood plasma in terms of ionic composition.

**Figure 8:**
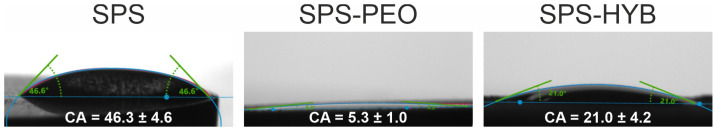
Image of SBF drops on samples with different types of surface treatment.

For uncoated samples, CAs values decrease by approximately three times, while for the SPS-PEO-treated sample, CAs values remain within the error (Figure 8). It should be noted that during the wettability investigation of uncoated samples, significant gas formation was observed upon contact with a droplet. This is explained by the low corrosion resistance of magnesium and the heterogeneity of its material structure, including the presence of individual grains, which caused the droplet to spread more rapidly.

For SPS-PEO and SPS-HYB, the CAs values remained unchanged and within the margin of experimental error (5.3 ± 1.0° for PEO, and 21.0 ± 4.2°, respectively) (Figure 8). This indicates that the coatings have optimal wettability and pronounced hydrophilic properties.

### 3.5. Coatings Mechanical Properties

**Figure 9:**
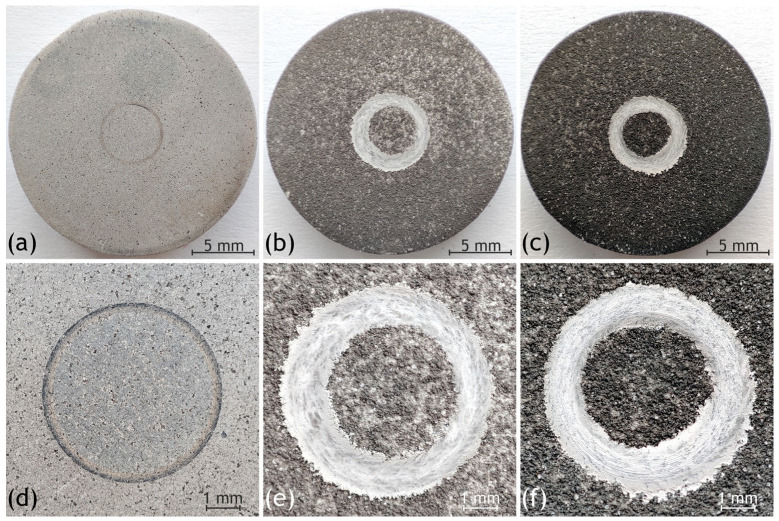
Appearance of wear tracks for the studied samples: bare SPS sample (**a**,**d**), with PEO coating (**b**,**e**), and with hybrid coating (**c**,**f**).

Evaluation of tribomechanical properties was performed by determining wear resistance and friction coefficient. Uncoated samples demonstrated the highest wear resistance (4.7 ± 2.3) × 10^−3^ mm/(N·m) and the lowest friction coefficient (Table 5). Magnesium acted as a dry lubricant, causing the friction coefficient to stabilize after just 10–15 cycles and remain virtually unchanged thereafter. Despite the presence of the calcium carbonate phase, the porosity of SPS samples was very low, and high-temperature pressing further densified the surface. Consequently, sample surface wear occurred slowly and uniformly compared to coated samples (Figure 9a,d).

The behavior of SPS-PEO and SPS-HYB samples was similar, despite the polydopamine layer on the latter. Both samples exhibited significantly higher wear rates (by an order of magnitude) compared to SPS samples, along with elevated friction coefficients (Table 5). The primary reason for this behavior is the porous structure of the PEO coating, particularly in its upper layers. During initial testing, the counterbody compresses these layers, resulting in the formation of a wide and deep wear track (Figure 9e,f). Wear particles from the coating then act as abrasives, accelerating wear rates and increasing the friction coefficient. The contact area between the counterbody and sample also affects the friction coefficient. As shown in Figure 9, coated samples have substantially larger contact areas compared to SPS samples. The polydopamine layer showed a negligible effect due to its minimal thickness and rapid degradation during initial revolutions, after which the PEO layer wear dominated.

Since none of the tested samples contained hard compounds, no wear of the corundum ball was observed.

## 4. Conclusions

In this study, the SPS parameters were optimized, and magnesium samples were produced for further investigation. The samples were fabricated from powdered magnesium material with a controlled concentration of CaCO_3_. A correlation was established between the proportion of CaCO_3_ and Mg in the mixture and the reduction in the absolute shrinkage of the material. An optimal percentage of 7 wt.% CaCO_3_ in the sintered material was identified.

Analysis of the roughness parameters of samples with PEO coatings using optical surface profilometry revealed an increase in roughness compared to the uncoated surface.

Studies of the hydrophobic properties of coatings revealed that all samples with hybrid coatings exhibited a decrease in contact angle and an increase in surface free energy compared to uncoated samples, a result of both the roughness of the PEO layer and the presence of a polydopamine film and water-soluble compounds within the coatings. Formation of coatings on samples resulted in an increase in surface free energy by more than 6 times (more than 70 mJ/m^2^), which has a positive effect on adhesion and cell growth rate.

The results of mechanical testing revealed that SPS-PEO samples possess a unique microstructure characterized by increased surface roughness and complex morphology, driven by the granular structure of the material and the presence of calcium carbonate inclusions. However, the higher wear rate and brittleness under fracture loads highlight the need for further optimization to balance wear resistance, surface morphology, and mechanical durability. These findings open prospects for the development of materials with enhanced performance characteristics, while also emphasizing the importance of considering trade-offs between structural and mechanical properties.

## Figures and Tables

**Table 1:** Description of the samples studied and corresponding designation.

Sample	Designation
Bare SPS-produced sample	SPS
PEO coating	SPS-PEO
PEO coating with vancomycin, menaquinone-7, zoledronate, PDA	SPS-HYB

**Table 2:** Roughness parameters (arithmetical mean height *S_a_* and root mean square height *S_q_*) of samples with different mass fractions of calcium carbonate.

Parameter	CaCO_3_ Content in SPS-Sample, wt.%						
	0	1	2	3	5	7	10
SPS							
*S_a_* (µm)	1.6 ± 0.1	1.7 ± 0.1	1.7 ± 0.1	1.9 ± 0.1	1.9 ± 0.1	1.8 ± 0.1	1.7 ± 0.1
*S_q_* (µm)	2.0 ± 0.1	2.1 ± 0.1	2.2 ± 0.2	2.3 ± 0.2	2.4 ± 0.2	2.4 ± 0.4	2.2 ± 0.5
SPS-PEO							
*S_a_* (µm)	4.8 ± 0.4	5.2 ± 0.1	5.5 ± 0.8	5.3 ± 0.1	6.4 ± 0.5	6.4 ± 0.6	5.9 ± 0.3
*S_q_* (µm)	6.3 ± 0.6	6.9 ± 0.4	7.1 ± 1.0	6.8 ± 0.1	8.2 ± 0.7	8.3 ± 0.9	7.6 ± 0.3

**Table 3:** Elemental composition of the PEO coating.

Element	Content of the Element, wt.%
P	29.4
Ca	28.5
Mg	21.1
Na	12.6
Si	5.9
Other	2.5

**Table 4:** Values of contact angles and free surface energy for SPS samples.

Sample	*θ*, °	*γ*_s_^d^, mJ/m^2^	*γ*_s_^p^, mJ/m^2^	*γ*_s_, mJ/m^2^
	H_2_O			
SPS	123.8 ± 3.1	12.85 ± 1.63	0.04 ± 0.01	12.89 ± 1.64
SPS-PEO	6.1 ± 0.6	50.19 ± 0.12	30.62 ± 0.09	80.81 ± 0.22
SPS-HYB	26.9 ± 4.1	49.22 ± 0.60	25.57 ± 1.68	74.79 ± 4.39

**Table 5:** Wear of the coatings.

Sample	Friction Coefficient μ	Wear (mm/(N m))
SPS	0.15 ± 0.01	(4.7 ± 2.3) × 10^−3^
SPS-PEO	0.75 ± 0.04	(4.6 ± 1.0) × 10^−2^
SPS-HYB	0.73 ± 0.05	(4.3 ± 1.2) × 10^−2^

**Figure A1:**
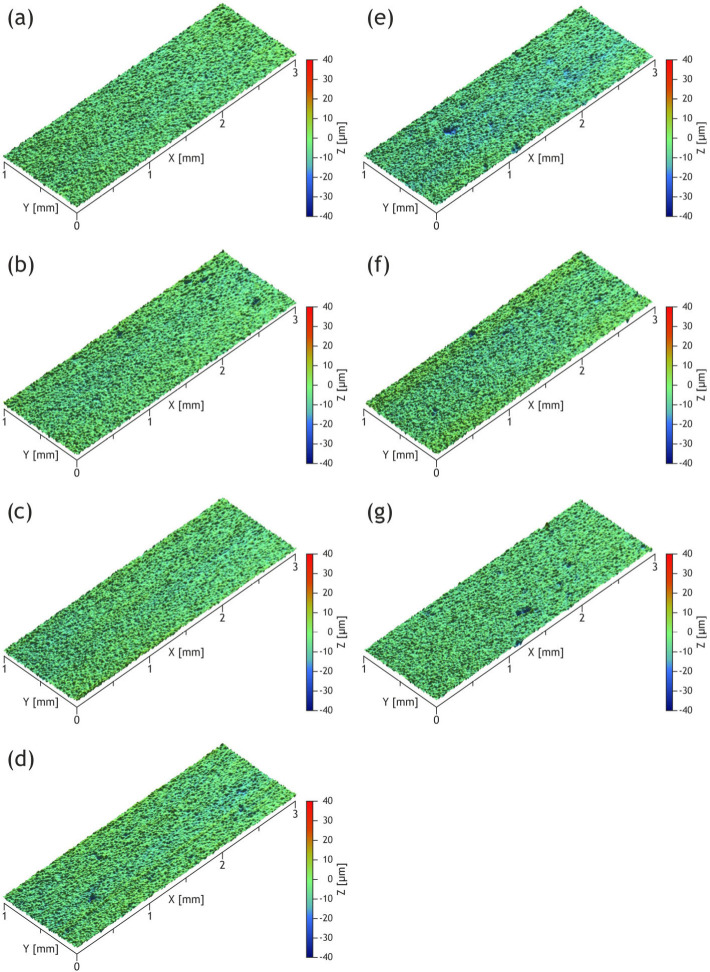
3D surface profiles for SPS samples with the following CaCO_3_ concentrations: (**a**)—0 wt.%, (**b**)—1 wt.%, (**c**)—2 wt.%, (**d**)—3 wt.%, (**e**)—5 wt.%, (**f**)—7 wt.%, (**g**)—10 wt.%.

**Figure A2:**
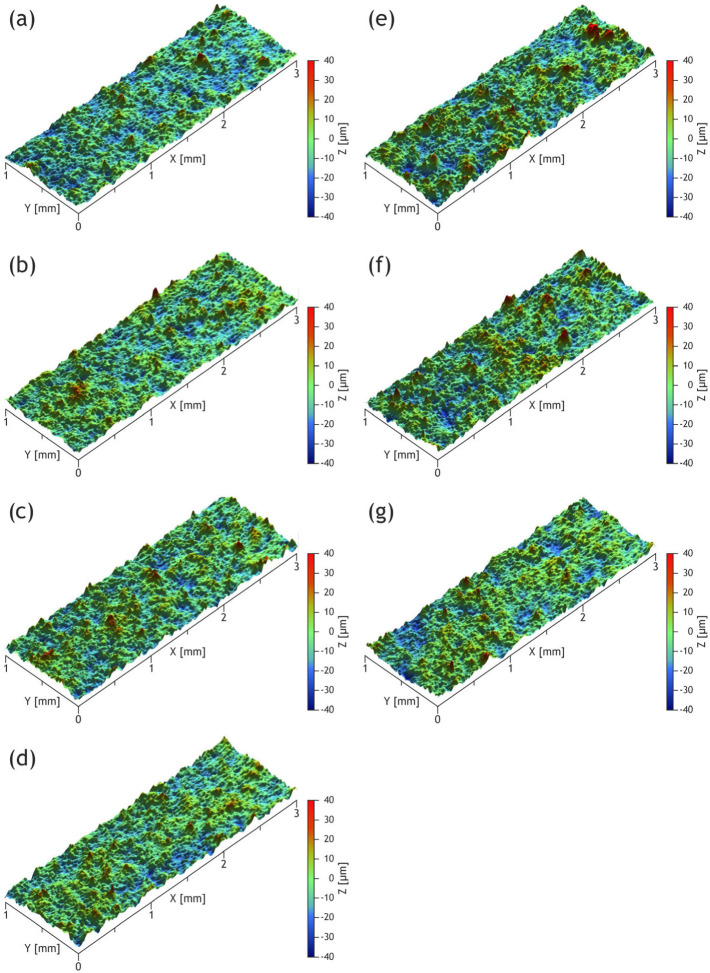
3D surface profiles of SPS-PEO samples with the following CaCO_3_ concentrations: (**a**)—0 wt.%, (**b**)—1 wt.%, (**c**)—2 wt.%, (**d**)—3 wt.%, (**e**)—5 wt.%, (**f**)—7 wt.%, (**g**)—10 wt.%.

**Figure A3:**
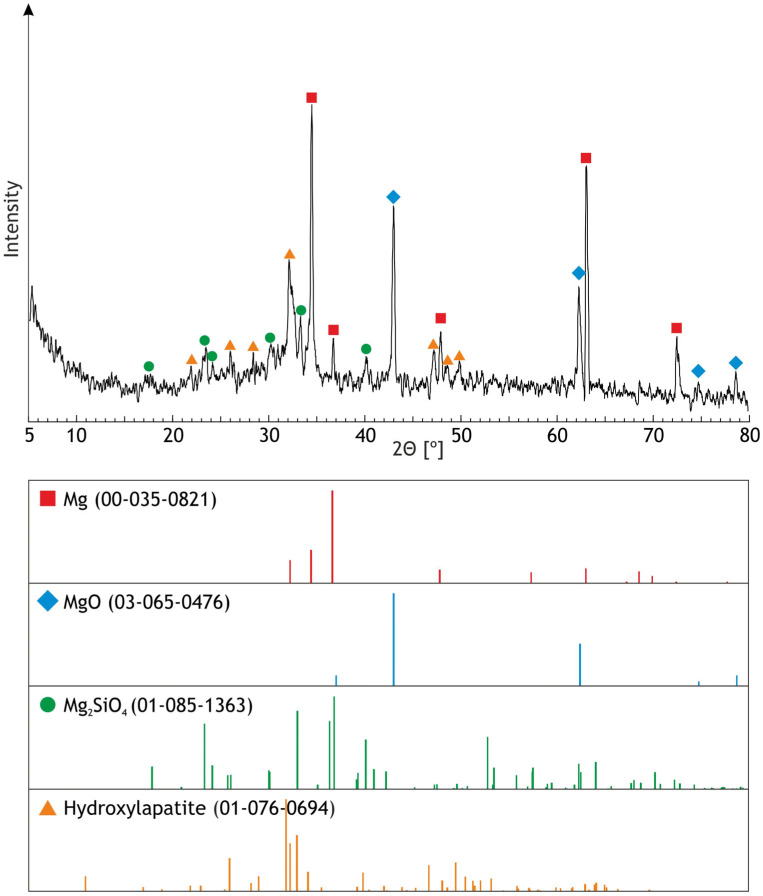
XRD pattern of the SPS-PEO sample.

## Data Availability

The data presented in this study are available on request from the corresponding author.

## Abbreviations

The following abbreviations are used in this manuscript:

IAIs	Implant-associated infections
SPS	Spark plasma sintering
PEO	Plasma electrolytic oxidation
HA	Hydroxyapatite
MK-7	Menaquinone-7 (Vitamin K2)
PDA	Polydopamine
HYB	Hybrid
XRF	X-ray fluorescence
XRD	X-ray diffraction
SEM	Scanning electron microscopy
EDS	Energy dispersive spectroscopy
OSP	Optical surface profiling
SBF	Simulated body fluid
SFE	Surface free energy
OWRK	Owens-Wendt-Rabel-Kaelble method
CA	Contact angle
IR	Infrared Spectroscopy
XPS	X-ray photoelectron spectroscopy

## Appendix A

### Appendix A.1

For initial samples with different calcium carbonate contents, the *Sa* and *Sq* parameters exhibit similar values. Upon analysis of the presented data, there is an increase in peak heights and valley depths, which is associated with an increase in the size and quantity of valleys (Figure A1).

This behavior of the parameters is attributed to the etching of calcium carbonate during the surface preparation and standardization process. At the same time, the maximum depth value was observed at a concentration of 3% by weight (Figure A1).

### Appendix A.2

An analysis of the roughness parameters of the samples with PEO coatings indicates an increase in the surface roughness compared to the samples without coating (Figure A2).
